# Tropical land carbon cycle responses to 2015/16 El Niño as recorded by atmospheric greenhouse gas and remote sensing data

**DOI:** 10.1098/rstb.2017.0302

**Published:** 2018-10-08

**Authors:** Emanuel Gloor, Chris Wilson, Martyn P. Chipperfield, Frederic Chevallier, Wolfgang Buermann, Hartmut Boesch, Robert Parker, Peter Somkuti, Luciana V. Gatti, Caio Correia, Lucas G. Domingues, Wouter Peters, John Miller, Merritt N. Deeter, Martin J. P. Sullivan

**Affiliations:** 1 School of Geography, University of Leeds, Leeds, UK; 2 NCEO, NERC National Centre for Earth Observation, Michael Atiyah Building, University of Leicester, Leicester, UK; 3 LSCE, L'Orme des Merisiers, Bat. 701, Point courrier 129, Gif sur Yvette Cedex, France; 4 Institute of Geography, University of Augsburg, Augsburg, Germany; 5 Department of Physics and Astronomy, University of Leicester, Leicester, UK; 6 INPE, Sao Jose dos Campos, Brazil; 7 Wageningen Universiteit en Researchcentrum, Wageningen, Gelderland, The Netherlands; 8 NOAA/Earth System Research Laboratory/Global Monitoring Division, Boulder, CO, USA; 9 NCAR Atmospheric Chemistry Division, Boulder, CO, USA

**Keywords:** carbon cycle, global warming, fire, tropical forests

## Abstract

The outstanding tropical land climate characteristic over the past decades is rapid warming, with no significant large-scale precipitation trends. This warming is expected to continue but the effects on tropical vegetation are unknown. El Niño-related heat peaks may provide a test bed for a future hotter world. Here we analyse tropical land carbon cycle responses to the 2015/16 El Niño heat and drought anomalies using an atmospheric transport inversion. Based on the global atmospheric CO_2_ and fossil fuel emission records, we find no obvious signs of anomalously large carbon release compared with earlier El Niño events, suggesting resilience of tropical vegetation. We find roughly equal net carbon release anomalies from Amazonia and tropical Africa, approximately 0.5 PgC each, and smaller carbon release anomalies from tropical East Asia and southern Africa. Atmospheric CO anomalies reveal substantial fire carbon release from tropical East Asia peaking in October 2015 while fires contribute only a minor amount to the Amazonian carbon flux anomaly. Anomalously large Amazonian carbon flux release is consistent with downregulation of primary productivity during peak negative near-surface water anomaly (October 2015 to March 2016) as diagnosed by solar-induced fluorescence. Finally, we find an unexpected anomalous positive flux to the atmosphere from tropical Africa early in 2016, coincident with substantial CO release.

This article is part of a discussion meeting issue ‘The impact of the 2015/2016 El Niño on the terrestrial tropical carbon cycle: patterns, mechanisms and implications’.

## 1. Introduction

**Figure 1.**
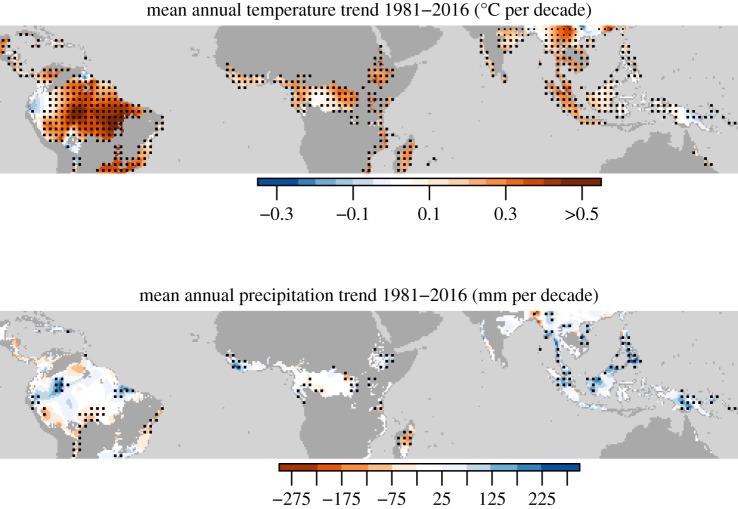
Climate trends for tropical and subtropical forest biome based on CRU (Climate Research Unit) TS 3.24 climatology [4]. Stippling denotes statistically significant trends.

Tropical forests play a vital role in the Earth system, hosting greater than 50% of global terrestrial biodiversity (e.g. [1]), storing two-thirds of global plant biomass (e.g. [2]) and regulating climate by virtue of their exchanges of carbon, water and energy with the atmosphere. They also play an important role in sustaining endangered fauna, and their continued presence is essential for preserving their rich biodiversity. More generally, tropical biomes are home to great cultural diversity and growing economies, and will need to support half the global population by 2050 [3]. Thus they have a large impact on livelihoods in these climates. The continued functioning and productivity of vegetation in the tropics is, however, dependent on its response to changing climatic conditions. The dominant climate change signatures across the tropics are rapid warming and an increase of extreme events, severe floods and anomalously dry conditions (figure 1 and e.g. [5]).

El Niño events may provide a test bed to examine tropical vegetation responses, likely to be dominated by the response of forests, to these increasingly higher temperatures, paralleled usually by drier than usual conditions. This is because during El Niño events strong positive temperature excursions tend to be spatially correlated with negative precipitation anomalies. These anomalies occur in tropical Southeast Asia, tropical South America and to a lesser extent tropical West Africa and southern Africa (roughly below 10° S, e.g. [6,7]).

It has been known since at least the 1970s that El Niño events co-occur with periods of anomalously large atmospheric CO_2_ growth rates [8]. There is not only a strong correlation between the El Niño Index (in essence atmospheric sea surface pressure difference between Darwin and Tahiti in the tropical Pacific) and global atmospheric CO_2_ growth rate anomalies but also a slightly weaker correlation with tropical land surface temperature anomalies (e.g. [9]). The mechanism causing the correlation with temperature is not entirely clear. One component is increased biomass burning. It has further been argued that the effect of water limitation on vegetation performance is important at the local scale, but temperature anomalies are more important at larger scales owing to cancelling effects [10]. The strong correlation between atmospheric growth rate anomalies and El Niño Index suggests that the variation of tropical land carbon uptake and release contributes prominently to anomalous atmospheric CO_2_ growth during positive El Niño phases. Nonetheless, ocean air–sea gas exchange does also play a role. Measurements of this process in the tropical Pacific reveal that during El Niño outgassing in the tropics is reduced, i.e. ocean carbon pool response is in the opposite direction to land carbon pools [11]. This is because the tropical Pacific thermocline upward tilt towards South America is reduced during El Niño phases of ENSO (El Niño Southern Oscillation), which hinders upwelling of carbon-rich waters along the tropical South American west coast and thus carbon efflux from the sea to the atmosphere is reduced. The decrease over a full El Niño period for the 1997/98 event has been estimated using ocean data to be 0.6 ± 0.1 PgC ([11,12]). Recent estimates of global air–sea gas exchange based on air–sea partial pressure difference measurements and gas exchange parameterization by Feely *et al*. [13] suggest a smaller anomaly over the 2015/16 period of the order of 0.1–0.2 PgC.

Similar to the other contributions to this volume, we attempt to analyse whether, and to what extent, the response of tropical land vegetation during the 2015/16 El Niño event is different from responses during previous similar events, and thus may be a harbinger of future responses not just to climate oscillations but climate variation on top of a rapidly increasing temperature background. There have been, for example, reports indicating that increased temperatures independent from El Niño have an effect on fire probability in the tropics [14]. There have also been reports indicating that dry seasons may get drier across the tropics [15]. It is not clear what the effect of tropical vegetation productivity may be, given both stimulating (rising CO_2_) and limiting (e.g. the increase in leaf–air water vapour pressure difference) factors. The overarching theme of this article is thus whether the 2015/16 events reveal signs of anomalously increasing vegetation strain.

Our analysis is based primarily on a large-scale atmospheric approach that as the main tool uses an inverse model of atmospheric transport (INVICAT [16]) to extract information about the surface CO_2_ exchange between land vegetation and atmosphere contained in spatio-temporal variations of atmospheric CO_2_. Our approach is helped by new data from tropical South America measured by INPE (Instituto Nacional de Pesquisas Espaciais), Sao Jose dos Campos, Brazil. We focus on the inter-annual variation of fluxes, which should be more robustly estimable than absolute flux magnitudes. To put our results into context we relate the fluxes to climate controls, and to distinguish processes, to some extent, we employ solar-induced chlorophyll fluorescence and atmospheric carbon monoxide measured from space. We aim to address the following questions: How anomalous is the global CO_2_ flux anomaly? What are the climate deviations/excesses on land? Where and when do flux anomalies occur and how large are they? How much is due to fire and under what conditions? How much is due to reduction in primary production versus changes in respiration? And finally, are there signs of land vegetation responses outside the usual El Niño patterns given the unprecedented temperatures during the 2015/16 event?

## 2. Data and methods

### (a) Estimating atmospheric growth rate anomalies

A possible approach to estimate atmospheric CO_2_ growth rate anomalies Δ*g*, suggested to our knowledge first by Jones *et al*. [17], is as follows:2.1
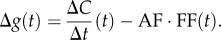
Here *C* is the atmospheric carbon content (in the form of CO_2_), *t* is time, FF is the global emissions from fossil fuel burning and cement manufacture, and AF is the long-term mean airborne fraction, the ratio of the annual atmospheric carbon growth rate and fossil fuel emissions. Thus AF · FF(*t*) is the expected average increase of atmospheric carbon growth rate for given fossil fuel emissions FF(*t*) in year *t*. Fossil fuel emission estimates used here are from Boden *et al*. [18], which are based on energy statistics and the observed atmospheric CO_2_ record (the Mauna Loa record) AF ≈ *ca* 0.55. The growth rate is calculated as the difference of annual means centred on 31 December/1 January (i.e. mean from 1 July to 30 June). As a sensitivity test, we have repeated this calculation using AF = 0.49 (mean over 1901–2015) with similar overall conclusions.

### (b) Carbon flux estimation from atmospheric CO_2_ patterns with inverse modelling of atmospheric transport

The most robust information provided by atmospheric CO_2_ concentration records is the global atmospheric inventory and how it changes over time. This reveals, for example, very clearly the well-known rapid increase of atmospheric CO_2_ over the last decades. In addition to the global information, the widespread surface station observation network, maintained by various groups and in particular NOAA/ESRL (electronic supplementary material, figure S1), exhibits spatio-temporal patterns that reflect regional-scale variation in CO_2_ exchange between the land surface and oceans with the atmosphere. Thus, in principle, these patterns should allow us to trace back the spatial distribution and strength of regional surface fluxes, provided the relationship between fluxes and the concentration patterns they cause can be established. This relationship involves the representation of the processes of atmospheric advection and dispersion, which can be estimated fairly well using numerical fluid dynamics models of the atmospheric flow (atmospheric transport models) (e.g. [19]). The relation to the actual atmospheric flow is established by using wind and cloud convection transport fields derived from regular observations of the state of the atmosphere for the purpose of weather prediction. Flux estimation reduces then to a least-squares minimization problem of the difference between a linear combination of concentration fields resulting from localized fluxes in space and time sampled at the same time and location as the observations and the actual observations. This problem turns out to be poorly constrained by the number of available *in situ* measured data and thus a possible approach is to instead optimally combine a set of prior flux ‘guesses' **f**_p_ with the flux estimates that replicate concentration data most closely [20], i.e. to minimize2.2
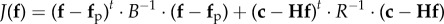
with respect to **f**. **B** is the *a priori* flux error covariance matrix, **c** is a vector containing the observed atmospheric concentrations and **H** is the transport-model-calculated matrix, which relates surface fluxes to the atmospheric concentration signal they cause at the sampling sites. This approach solves for small deviations from a prescribed flux model. For this problem, an explicit expression for the posterior flux error covariance matrix ***A***_post_ can be derived [21]: ***A***_post_ = [***H***^t^ · ***R***^−1^ · ***H* + *B***^−1^]^−1^.

We show here the results from such an approach based on the inverse of the atmospheric transport model TOMCAT [22]. We resolve fluxes monthly and spatially on a grid 5.6° × 5.6° longitude by latitude and the model is forced by ERA-Interim meteorology. The prior flux model includes three components: (i) annually changing fossil fuel emissions, (ii) monthly net land gains or losses which do not change from year to year, based on the CASA (Carnegie–Ames–Stanford) land biosphere model (average climatology for 2003–2011), and (iii) air–sea fluxes. The CASA model estimates primary productivity as the product of solar photosynthetically active radiation (PAR), land vegetation chlorophyll content (estimated using Normalized Difference Vegetation Index (NDVI) measured from space) and a light use efficiency. Respiration is estimated using a carbon cycle model that includes soils [23].

We have chosen annually repeating and balanced land vegetation–atmosphere CO_2_ flux prior estimates because our interest is in extracting the information on inter-annual variations contained in atmospheric data. For our prior estimates of air–sea fluxes, we treat separately the fluxes associated with the pre-industrial carbon cycle (two hemispherical loops with CO_2_ outgassing in the tropics and CO_2_ uptake at high latitudes) and uptake of carbon induced by the anthropogenic perturbation of atmospheric CO_2_ (with fluxes steadily increasing and located primarily in the northern Atlantic and Southern Ocean) [24,25]. For the former, we use the monthly resolved climatology based on air–sea partial pressure differences and an air–sea gas exchange coefficient parameterization, compiled by Takahashi *et al*. [26], to which we add a constant and spatially uniform flux such that the fluxes are globally in balance on an annual basis. For the latter, we use the spatial air–sea flux pattern of Khatiwala *et al*. [25] (their figure 1*b*), which we scale with global net ocean uptake taken from the Global Carbon Project analysis [27]. This approach leads to improved *a posteriori* data model fits compared with inversions which use air–sea flux prior compilations based on interpolation algorithms of air-to-sea partial pressure measurement differences alone (specifically [26]). The minimization of *J*(**f**) is done using a quasi-Newtonian method (L-BFGS implemented in M1QN3 minimizer) with gradients calculated with the adjoint of the TOMCAT atmospheric transport model, ATOMCAT [16]. We assume a prior flux uncertainty of 200% per grid cell and we assume that there is no flux error correlation given the comparably coarse resolution. Atmospheric data are from 81 sites mainly measured by NOAA/ESRL and include in addition the planetary boundary layer (PBL) mean (below 2500 m) and the free troposphere mean (above 2500 m) of vertical profile data in the Amazon measured by INPE, Sao Jose dos Campos, Brazil (e.g. [28]) (electronic supplementary material, figures S1 and S2). Observational data have an uncertainty of 1 ppm plus an estimate of representation error derived by averaging the absolute prior concentration variation between the model grid cell containing the measurement location and the surrounding grid cells. This leads to overall observational uncertainties of between 1 and 6 ppm, depending on the measurement location. To assess the influence of the Amazon vertical profile data, we have also performed an inversion without these data. The effect of including the data is to reduce the magnitude of flux anomalies while the timing and location of anomalies are not affected much (see §3).

### (c) Gravity anomalies as an indicator of vegetation water stress

One cause of plant water stress is negative deviations (or anomalies) of the abundance of soil water (or soil water content) from the climatological mean representative for a region. A proxy for soil water content over large spatial scales can be measured from satellites because large-scale land surface water content anomalies cause Earth gravity anomalies. Such gravity anomalies are being estimated from space by the twin satellite mission GRACE (Gravity Recovery and Climate Experiment [29]), with the satellites following each other closely on a polar orbit. Instruments on the satellites measure the distance between the two satellites, which increases when the front satellite is approaching a positive gravity anomaly and decreases again once the front satellite has passed the anomaly and the rear satellite is approaching the anomaly. To confirm the realism of the gravity anomaly data measured from space, we compare gravity anomaly anomalies with precipitation anomalies measured by TRMM (Tropical Rainfall Measuring Mission [30]; electronic supplementary material, figure S3). The signatures of the two data types are very consistent (taking into account that gravity anomalies are to first order equal to cumulative precipitation anomalies). To calculate monthly gravity anomaly anomalies, we subtract monthly mean values calculated using the full 2002–2016 record from the continuous record of monthly mean values.

### (d) Estimation of fire carbon release to the atmosphere from remotely sensed air column carbon monoxide inventories

We use daytime CO air column inventories estimated from MOPITT radiometer data on the TERRA satellite [31] to estimate carbon emissions from fires. To do so, we first estimate carbon monoxide fluxes from monthly CO air column anomalies. We then convert the carbon monoxide fluxes to carbon fluxes, assuming they are from fires, by multiplying the carbon monoxide fluxes with a biomass burning emission ratio of 1/74 ((ppm CO_2_)/(ppb CO)) [32] although this ratio may vary with the type of fire. The MOPITT CO record we are using is v. 6 (L3V95.2.3) [33]), which covers March 2000 to December 2016. This version uses both thermal infrared (TIR) and near-infrared (NIR) radiances and so, compared with the other two MOPITT products (TIR-only and NIR-only), it provides the maximum sensitivity to surface-level CO. Nonetheless, because of the non-uniform weighting function of the retrievals, column content estimates may contain a bias (an underestimate of column CO if signals are concentrated to the lower troposphere) (electronic supplementary material, figure S5). The retrieval calculations include a time-invariant prior and so retrieved anomalies stem entirely from the radiometric data.

To estimate the CO flux *F* from atmospheric total column CO, we use the mass balance equation for a fixed volume *V*:
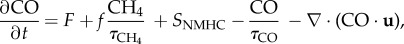
with *f* ≈ 0.85 the fraction of CH_4_ oxidized to CO, 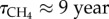 the lifetime of CH_4_ in the atmosphere, *τ*_CO_ ≈ 0.1 year the lifetime of CO in the atmosphere, *S*_NMHC_ the CO volume source due to the oxidation of non-methane hydrocarbons, and **u** the air flow velocity vector. We apply the equation to the total air volume above a fixed region to obtain a relation between a CO flux perturbation Δ*F* at the Earth's surface and the ΔCO anomalies it causes:
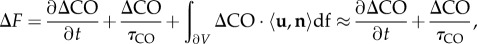
where **n** is an outwards directed unit normal vector orthogonal to a vertical wall ∂*V* surrounding the surface region of interest and df is an infinitesimal area element of *∂V*. The contribution of in- and outflows into and out of the air volume above the region is negligible if region boundaries are chosen such that ΔCO ≈ 0.

### (e) Solar-induced fluorescence

Photosynthesis is associated with fluorescence. A small fraction of solar radiation trapped by chlorophyll escapes instead of being used to fix CO_2_. This fraction is re-emitted into the atmosphere from the leaf at larger wavelengths (in the range of 670 and 800 nm, e.g. [34]) compared with the originally trapped radiation. Fluorescence has been shown to be related to productivity [35,36] and thus we use it here as a proxy for productivity. We specifically use here solar-induced fluorescence (SIF) data retrieved from GOSAT (Greenhouse Gases Observation Satellite [37]) measurements at 772 nm using the physically based retrieval technique described in Frankenberg *et al*. [38]. The bias correction procedure, which is an essential part of the post-retrieval processing, was performed using the European Space Agency Climate Change Initiative land cover maps [39]. GOSAT measurements over permanently non-vegetated areas (where zero fluorescence can be assumed) were identified using these maps in order to derive radiance-dependent calibration curves on a monthly basis. Based on these monthly curves, a two-dimensional spline interpolation was used along time and radiance dimensions to obtain the bias correction term for any given GOSAT sounding. This ensures that the time dependence of the instrument-related bias is taken into account. The retrievals are available for the period April 2009 to September 2016.

Monthly compilations of SIF retrievals exhibit missing values. To obtain sufficient data coverage we, therefore, calculate quarterly (three-monthly) means and similarly calculated anomalies for three-month periods. Despite lumping three months together, there are still pixels with no retrievals. To calculate quarterly anomalies we kept track of the number of existing retrievals on an individual pixel basis and averaged on a pixel basis. For the comparison of anomalies for specific areas and three-monthly periods, we calculated region mean anomalies including only those pixels for which retrievals exist.

## 3. Results

### (a) How anomalous is the global carbon cycle response to the 2015/16 El Niño event compared with earlier El Niño events?

**Figure 2.**
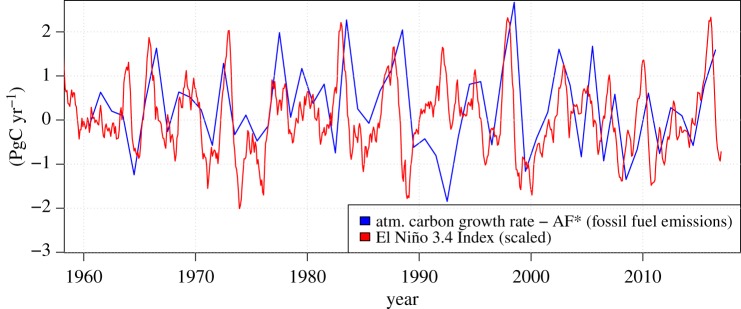
Global atmospheric CO_2_ growth rate anomalies Δ*g* estimated using equation (2.2) and El Niño 3.4 Index (obtained from http://www.cpc.ncep.noaa.gov).

The largest annual global atmospheric CO_2_ increase rates recorded with modern analytical tools (i.e. since 1959) occurred in 2015 and 2016, with values of 2.94 and 2.85 ppm, respectively (NOAA/ESRL, Boulder, Colorado, USA; ftp://aftp.cmdl.noaa.gov/products/trends/co2/co2_gr_gl.txt), which are slightly larger than the increase in 1998 (2.81 ppm). While of concern *per se*, to detect changes of El Niño land vegetation responses at the global scale, the nonlinearly increasing fossil fuel contribution to the atmospheric CO_2_ growth rate needs to be separated from other flux contributions. As explained in §2a, to achieve this, we assume a constant fossil fuel airborne fraction, which we subtract from the atmospheric carbon inventory growth rate (figure 2). The anomalies in 2015 and 2016 were positive and when summed up were approximately 1.7 PgC, with the 2016 anomaly being approximately twice as large as the 2015 anomaly. Including the reduction in CO_2_ outgassing from tropical oceans during positive El Niño episodes based on the air–sea flux estimates summarized in Feely *et al*. [13], then the total flux anomaly of global land carbon to the atmosphere was approximately 1.9–2.1 PgC over the 2 years (2015–2016).

A comparison with the 1997/98 El Niño anomaly reveals that the 2015/16 anomaly was not extraordinarily large, certainly of a smaller magnitude than the 1997/98 anomaly. This conclusion does not depend much on the period chosen to estimate the airborne fraction (see §2a). From a vegetation process response point of view, the 1997/98 anomaly is, however, somewhat unusual in that it includes a strong direct human-impact large-scale peat drainage component which in 1997/98 led to ‘catastrophic' peatland/peat forest fires and carbon release [40]. Thus part of the 1997/98 positive anomaly is unrelated to climate-induced variation in productivity and respiration of living vegetation or soil respiration in a strict sense. A noticeable indirectly El Niño-related aspect revealed by growth rate anomalies (figure 2) is the negative (land carbon uptake) anomalies from roughly 2008 onwards.

### (b) Temperature and soil water content anomalies

**Figure 3.**
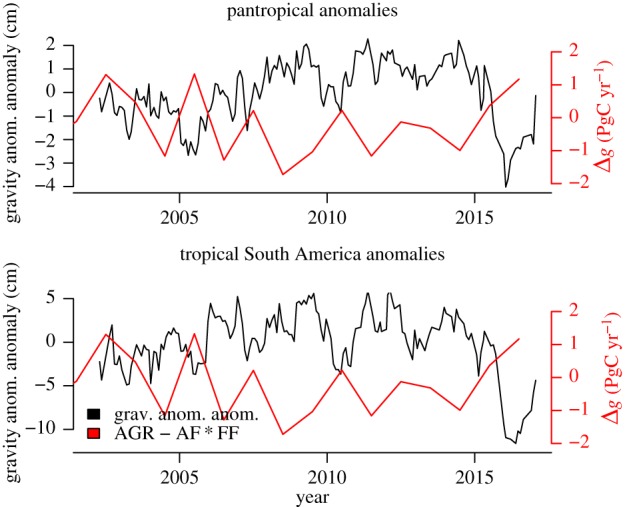
Tropical land gravity anomaly anomalies measured by the GRACE satellites and global CO_2_ growth rate anomalies Δ*g* estimated using equation (2.2).

In the spirit of using climate excursions associated with El Niño to examine tropical vegetation (primarily forest) response/sensitivity to elevated temperatures and drier than usual conditions, we briefly summarize here measures of vegetation stress related to climate. The first and primary measure is plant water stress caused by negative deviations (or anomalies) of the abundance of soil water (or soil water content) from the climatological mean representative for a region. We use here monthly gravity anomaly anomalies measured by GRACE (see §2c) as a proxy for soil water stress. Although these anomalies include both below- and above-ground water anomalies our use here as a vegetation water stress indicator is supported by anti-correlation between annual pan-tropical land/tropical South American land gravity anomaly anomalies and global atmospheric CO_2_ growth rate anomalies Δ*g* (see §2a) shown in figure 3 (Pearson *r* = −0.69 and −0.72, and *p* = 0.0046 and 0.0025, respectively).

**Figure 4.**
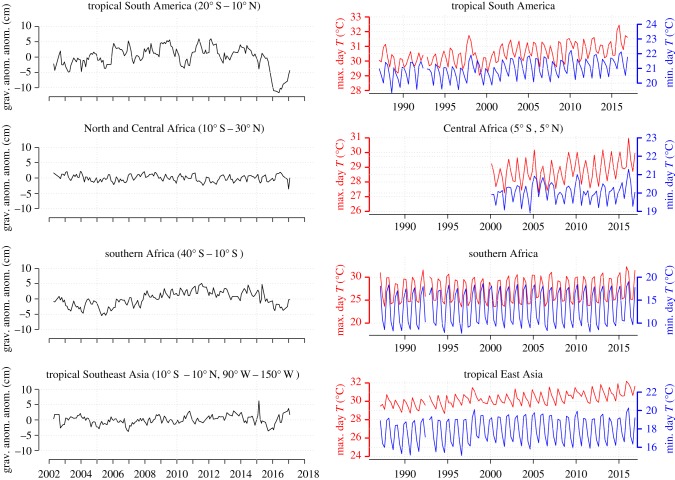
Land gravity anomaly anomalies and monthly means of daily minimum and maximum temperatures, respectively, for tropical land regions. Temperature data are from Climate Prediction Center (CPC), Global Land Surface Air Temperature Analysis [41].

The main features of vegetation water deficits during the 2015/16 El Niño period according to both gravity anomaly and precipitation data (figure 4; electronic supplementary material, figures S3 and S3b, S4) are as follows: (i) In the Amazon Basin, an east-to-west spreading and steadily increasing area with large water deficit, with this process starting at the beginning of 2015 (figure S3). The deficit reached its peak and covers the entire basin by the first quarter of 2016, with water deficit remaining high throughout the basin until the final quarter of 2016; the most pronounced negative precipitation anomaly occurred during the final quarter of 2015 all across the basin. (ii) In Africa a considerable water deficit developing south of roughly 10° S during the first three quarters of 2016, although the anomaly is not as strong as for Amazonia. The most pronounced precipitation anomaly for the region southward of 10° S occurred during the final quarter of 2015, i.e. a bit earlier than in the other two regions. A second notable feature is excessively hot conditions in the Congo Basin (west equatorial Africa; host to most of the remaining African humid forests) around February 2016, while according to gravity anomalies and precipitation estimated by TRMM there were no very clear indications of drought conditions but this will need further investigation. (iii) In tropical Southeast Asia strong negative precipitation anomalies and associated water stress during the second half of 2015. These three regions experienced strongly elevated temperatures nearly synchronously with the substantially drier than usual conditions, with peak temperatures all exceeding existing historical records (figure 4; electronic supplementary material, figure S3).

Among the three continents, the climate anomalies for the Amazon seem to be the strongest, with temperature and precipitation anomalies centred around the last three months of 2015 and first three months of 2016, and with the effects of precipitation anomalies on soil moisture lasting over nearly all of 2016, reflecting the time it takes for water deficits to propagate through the catchment (electronic supplementary material, figure S4).

Overall the observed climate anomalies are similar to the canonical El Niño patterns as described, e.g. by Dai & Wigley [42], with the tropical Asian precipitation anomaly being somewhat weaker. The possible exception is the Congo Basin, which was excessively hot during the first quarter of 2016.

### (c) Carbon fluxes estimated from atmospheric CO_2_ and inverse modelling of atmospheric transport

**Figure 5.**
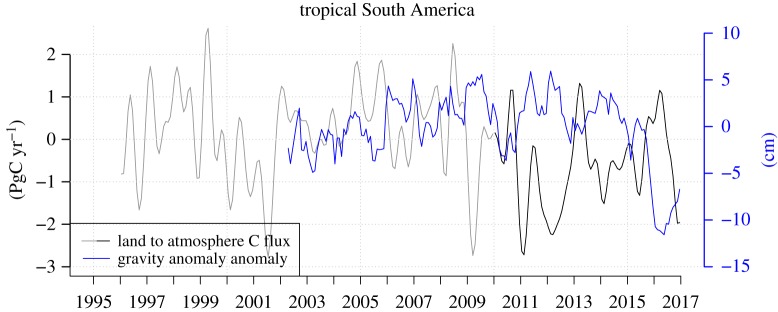
Tropical South America to atmosphere CO_2_ flux anomalies estimated with inverse modelling of atmospheric transport and atmospheric CO_2_ concentration observations (estimates indicated in black include vertical profile data available from 2010 onwards), and tropical South American gravity anomaly anomalies estimated by GRACE satellite mission.

**Figure 6.**
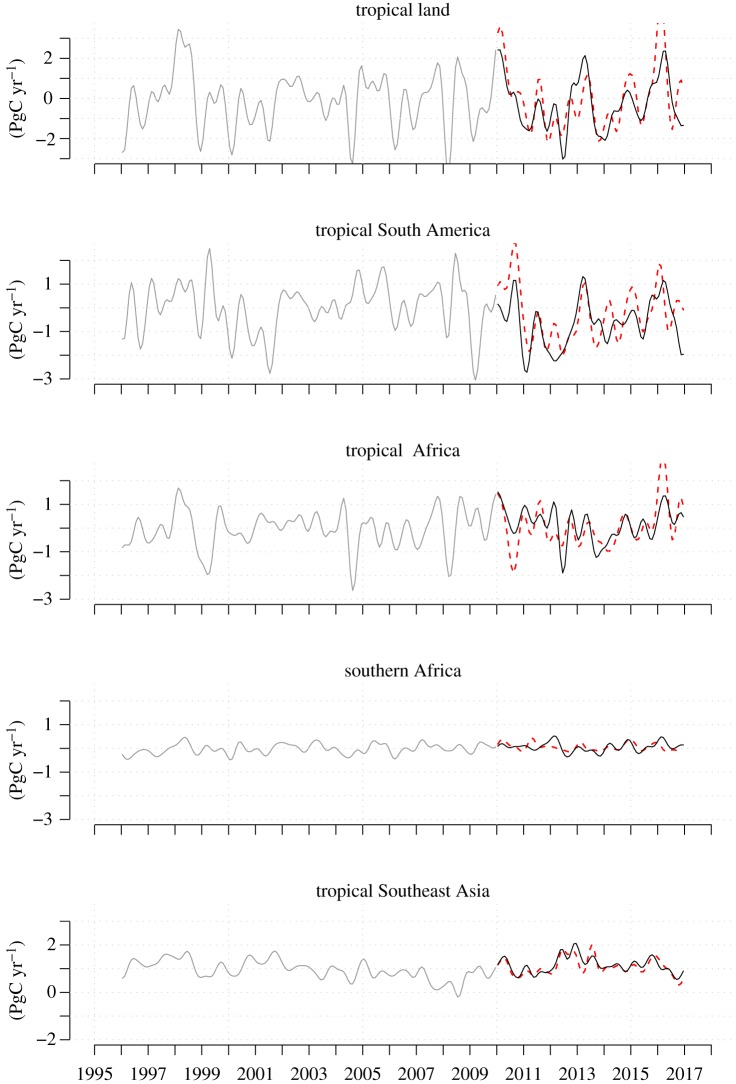
Time series of land-to-atmosphere carbon flux estimates (low-pass filtered) for tropical land regions. The portion for which Amazon vertical profile data are available and included in the atmospheric transport inversion calculations is coloured in black. Dashed lines show estimates that do not include tropical South American data. (Online version in colour.)

What do atmospheric inversion results suggest? Motivated by the relation between inter-annual variation of the global atmospheric CO_2_ growth rate and gravity anomaly anomalies on tropical land, we compare land CO_2_ flux anomalies with gravity anomaly anomalies for tropical South America as measured by the GRACE satellite mission (figure 5). The flux anomalies are calculated from the net flux estimates, which include all processes, also including in particular fossil fuel emissions. The main outstanding feature is the fairly close synchronicity of positive flux anomalies (fluxes to the atmosphere) with negative gravity anomaly anomalies and vice versa (Pearson *r* = −0.42 for monthly means, *p* < 10^−3^), which is consistent with the global record (figure 2). This result demonstrates the inversion's ability to detect and attribute expected flux anomalies from the atmosphere data. Splitting up the flux estimates by those regions with notable climate anomalies we find the following (figure 6). According to our calculations, four regions released significant amounts of carbon during the 2015–2016 period: tropical South America, tropical Africa, southern Africa and tropical East Asia. The losses from tropical South America and tropical Africa are similar in magnitude while losses from tropical East Asia and southern Africa are smaller (table 1). The timing of peak carbon losses differs between the regions, with a peak in October 2015 for tropical East Asia, February 2016 for southern Africa, February and March 2016 for tropical Africa, November to December 2015 and March to April 2016 for tropical South America.

**Table 1.** Tropical land carbon flux anomalies.

		net carbon flux to atmosphere	biomass burning carbon flux
region	period	(PgC)	(PgC)
tropical South America	Sep 2015 to June 2016	0.5 ± 0.3	0.05–0.1
tropical Africa	Nov 2015 to July 2016	0.6 ± 0.3	0.08–0.16
southern Africa	Jan 2016 to May 2016	0.2 ± 0.1	
tropical Southeast Asia	Sep 2015 to Dec 2015	0.2 ± 0.1	0.3–0.4

### (d) Disentangling processes contributing to flux anomalies

**Figure 7.**
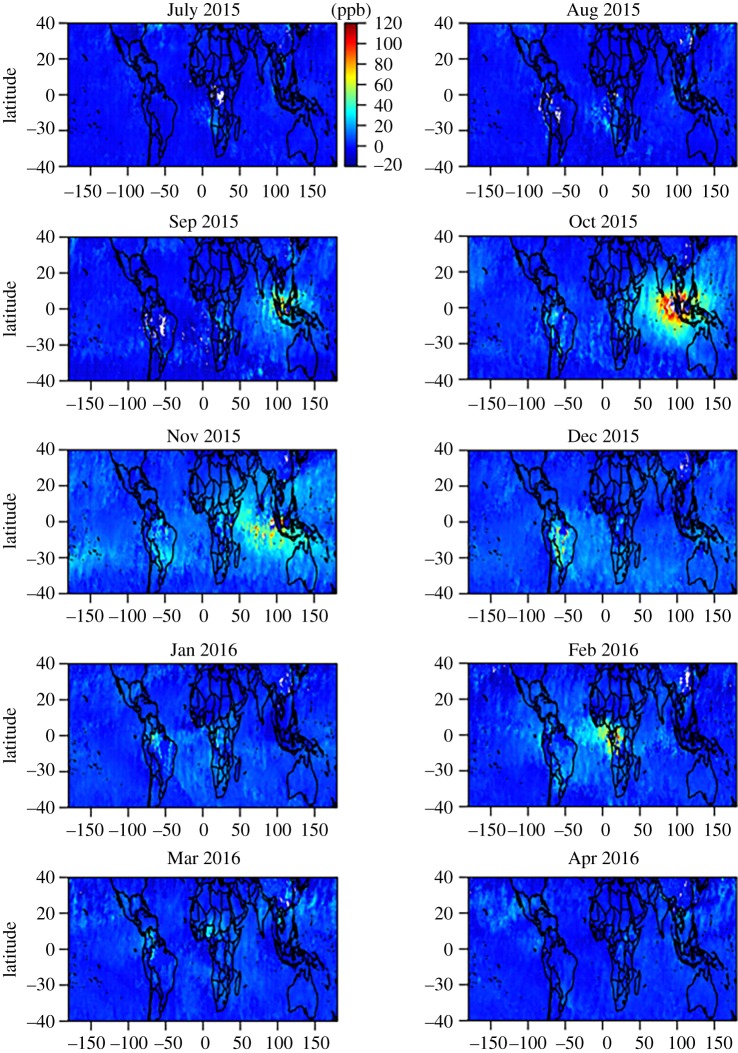
Total column carbon monoxide anomalies during 2015/16 of total air column carbon monoxide measured from space (MOPITT [33]).

**Figure 8.**
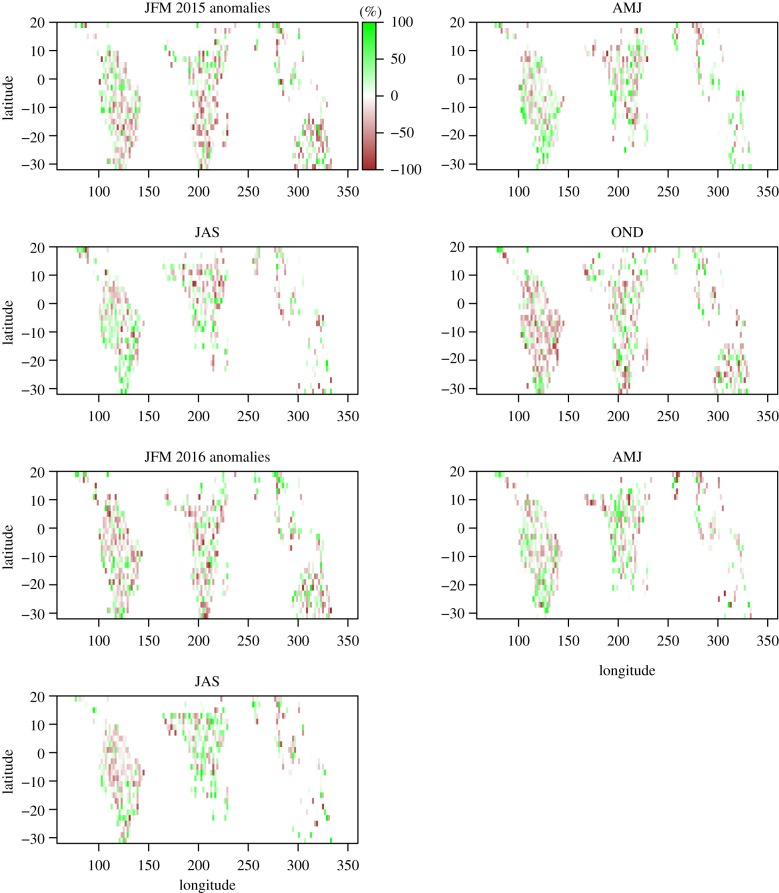
Solar-induced fluorescence anomalies (measured from GOSAT satellite [37] and based on retrievals at 772 nm). JFM, January, February and March, etc.

CO_2_ estimates based just on atmospheric CO_2_ concentration data and inversion of atmospheric transport provide net fluxes but cannot discern between the different underlying processes, such as biomass burning, or changes in vegetation productivity and respiration processes (e.g. by living trees/vegetation and/or dead organic matter in soils). Here, in addition, we analyse information from atmospheric total column carbon monoxide (CO) for the period 2000–2017, retrieved from the MOPITT (Measurements of Pollution in the Troposphere) radiometer on the NASA TERRA polar orbiting satellite, as an indicator of release of carbon via fire (figure 7) [31] and solar-induced fluorescence (SIF) retrieved from GOSAT radiance measurement as an indicator of land vegetation productivity and covering the period April 2009 to September 2016 (figure 8).

Monthly CO air column anomalies, ΔCO, from MOPITT reveal a strong two to three-month-long release pulse from tropical East Asia centred on October 2015, followed by release from tropical South America during November and December 2015, and a pulse from the Congo Basin in February 2016 (figure 7). We apply the mass balance approach described in §2d to estimate CO flux anomalies from the three regions for which the MOPITT CO retrievals exhibit distinct positive anomalies (figure 7) with region boundaries chosen such that ΔCO ≈ 0 along the boundaries (figure 7; electronic supplementary material, table S1). We find quite small carbon emissions from biomass burning from tropical South America and Africa (0.1–0.2 PgC each) and larger emissions from tropical East Asia (0.3–0.4 PgC) (table 1).

The main feature revealed by monthly SIF anomalies (figure 8) is a strong decrease over tropical South America, particularly during October to December 2015 and to lesser extent subsequent months. When spatially integrated over tropical South America, the decrease during October to December 2015 is approximately 20%. To obtain a rough estimate of the associated decrease in carbon uptake, we use an estimate of tropical South American vegetation annual productivity (gross primary productivity, GPP) estimated by Jung *et al*. [43] based on CO_2_ flux measurement between the atmosphere and forest canopies. The annual productivity of tropical South American vegetation according to Jung *et al*. [43] is approximately 18 PgC. According to SIF, we obtain a three-month reduction in productivity in this region of 20% and thus obtain a reduction of carbon uptake of approximately 0.9 PgC during this quarterly period. Given limited evaluation of the SIF–GPP relationship in the tropics, this estimate needs to be taken with some caution. For the other quarterly periods, fluorescence anomalies are lower and signs of change less coherent across large regions.

### (e) Discussion and conclusion

From a climate perspective, the outstanding development in the tropics on land over the past decades is rapid warming. The 2015/16 El Niño adds a positive temperature anomaly on top of this already rapidly warming ‘background'. It thus provides a natural sensitivity experiment of tropical forest vegetation subject to high temperatures in the future. Because of the already elevated background temperatures, vegetation responses might be more severe compared with responses observed during previous El Niño events. Based on just the global atmospheric CO_2_ record, we do not find any obvious sign of anomalously large carbon release during the 2015/16 El Niño compared with El Niño events in the past. This does not exclude compensating effects at continental to regional scales. At these scales, there is a strong spatial correlation between positive temperature peaks and negative soil water anomalies diagnosed via gravity anomaly anomalies. Soil water content anomalies are expected to be related to land carbon exchange anomalies, which are indeed consistent with a correlation of anomalies on land with the global atmospheric CO_2_ record. By far the largest negative near-surface water content anomalies occurred in the Amazon Basin during the final quarter of 2015 and the first quarter of 2016 for the available record (2002–2017). Negative anomalies also occurred during an approximately two month period in tropical East Asia centred on October 2015 and Southern Africa during the first two quarters of 2016. We estimated continental scale CO_2_ flux values based on atmospheric concentration data from surface station networks complemented by vertical profile data in the Amazon. The results of these calculations should be taken with some caution, because uncertainty in model transport can lead to biased flux results (e.g. [44]). Despite this, the inter-annual variability may still be robust because transport modelling biases will affect all years in a similar way, meaning that correlations with environmental variables can still be reliable. In our study, high correspondence between tropical South American flux anomalies and negative precipitation anomalies gives some confidence in the results, as well as the covariation in time with climate anomalies and atmospheric CO anomalies. We find roughly equal net flux anomalies from the Amazon and tropical Africa of around 0.5 PgC each, and somewhat smaller positive flux anomalies from tropical East Asia and southern Africa. According to atmospheric CO anomalies, our analysis attributes anomalous carbon release from tropical East Asia to fires peaking in October 2015, while consistent with fluorescence data from space, biomass burning played a smaller role in the Amazon where the flux anomaly was reasonably consistent with the downregulation of primary productivity during peak negative water anomaly (final quarter of 2015 and first quarter of 2016). The one feature in our results that seems somewhat unexpected, as this is not usually a region considered to be affected significantly by El Niño, is the anomalous flux from tropical Africa coincident with substantial CO release from the Congo Basin, during the first quarter of 2016. Our estimate of CO_2_ released by fires from tropical Africa explains one-third of the flux anomaly estimated by the atmospheric transport inversion. Although there was a weak water deficit diagnosed by GRACE, which may have caused an anomalous decrease in productivity, SIF data do not give strong support to this mechanism. Thus, in addition to changes in productivity, enhanced heterotrophic respiration may have contributed also to this signal.

Finally, we examine how our results summarized together with main controls in table 2 compared with the recent analyses of Liu *et al*. [45] based primarily on satellite data. For the comparison, it is important to realize that the Liu *et al*. study calculated anomalies with reference to flux estimates from the year 2011, a La Niña year. It is well established that during La Niña years global CO_2_ growth rate anomalies are strongly negative. Thus Liu *et al*.'s point of reference is quite different from ours. Taking this into account, our results are similar with the exception of Africa. At the pan-tropical level, Liu *et al*. [45] estimate a difference of flux from land to atmosphere of 2.5 PgC for the period May 2015 to April 2016 compared with January 2011 to December 2011. Their specific choice is likely motivated by maximum positive anomalies. If we use a similar criterion and thus use the period July 2015 to June 2016, we find a difference of 2.4 PgC. With regards to tropical Africa, in contrast to Liu *et al*. [45], we find a substantial carbon loss from tropical Africa at the same time as the very strong heat peak in the Congo Basin (the beginning of 2016) when a clear CO anomaly also occurred. Our rough biomass burning estimate cannot explain this result on its own—thus some downregulation of tropical forest productivity or enhanced respiration would be needed to explain it. In comparison with Liu *et al*. [45], our inverse calculations also attribute less carbon release from southern Africa during the 2015–2016 El Niño period.

**Table 2.** Chronology and magnitude of carbon flux anomalies (Cflx) (sign convention based on a land vegetation perspective, i.e. anomalous carbon loss to the atmosphere has a negative sign while anomalous uptake has a positive sign), climate (‘H_2_O’: soil water status, ‘*T*’ temperature) and process diagnostics: carbon monoxide (CO) and solar-induced fluorescence (SIF). Symbols indicate the existence of positive (+) and negative (−) anomalies and the number of symbols the strength of the anomalies. JFM etc. indicate the three-monthly intervals.

	tropical South America					tropical Africa					southern Africa					tropical East Asia				
	H_2_O	*T*	Cflx	CO	SIF	H_2_O	*T*	Cflx	CO	SIF	H_2_O	*T*	Cflx	CO	SIF	H_2_O	*T*	Cflx	CO	SIF
2015																				
JFM																				
AMJ																				
JAS	−	++										+				−−		−		
OND	−−−	++	−−	+	−−−						−−	++				−−	+	−	+++	
2016																				
JFM	−−−	+	−−−			?	+++	−−−	++		−−		−−				++			
AMJ	−−	+									−−				n.a.					
JAS	−				−						−−				n.a.					
OND											−									

Symbols indicate the existence of positive (+) and negative (−) anomalies and the number of symbols the strength of the anomalies.

## Supplementary Material

Supplementary material

## Supplementary Material

Figure S2

## Supplementary Material

Figure S3

## Supplementary Material

Figure S4A and B

## Data accessibility

Additional data are provided as electronic supplementary material.
