# Supplementary material for: Tropical land carbon cycle responses to 2015/16 El Niño as recorded by atmospheric greenhouse gas and remote sensing data
Source: Philos Trans R Soc Lond B Biol Sci. 2018 Oct 8;373(1760):20170302. doi: 10.1098/rstb.2017.0302 (PMC6178440; doi:10.1098/rstb.2017.0302)
Supplement: Supplementary material [file rstb20170302supp1.docx]

**Supplementary Materials**

Table S1 Domains used for spatial integration of air column CO anomalies.

| Region | Latitude | Longitude |
| --- | --- | --- |
| Tropical South America | 20.5 S,...,19.5 N | 80.5 W,..., 20.5W |
| Tropical Africa | 20.5 S,...,19.5 N | 20.5 W,...39.5E |
| Tropical Asia | 20.5S,...,19.5 N | 49.5 E, ..., 149.5 E |


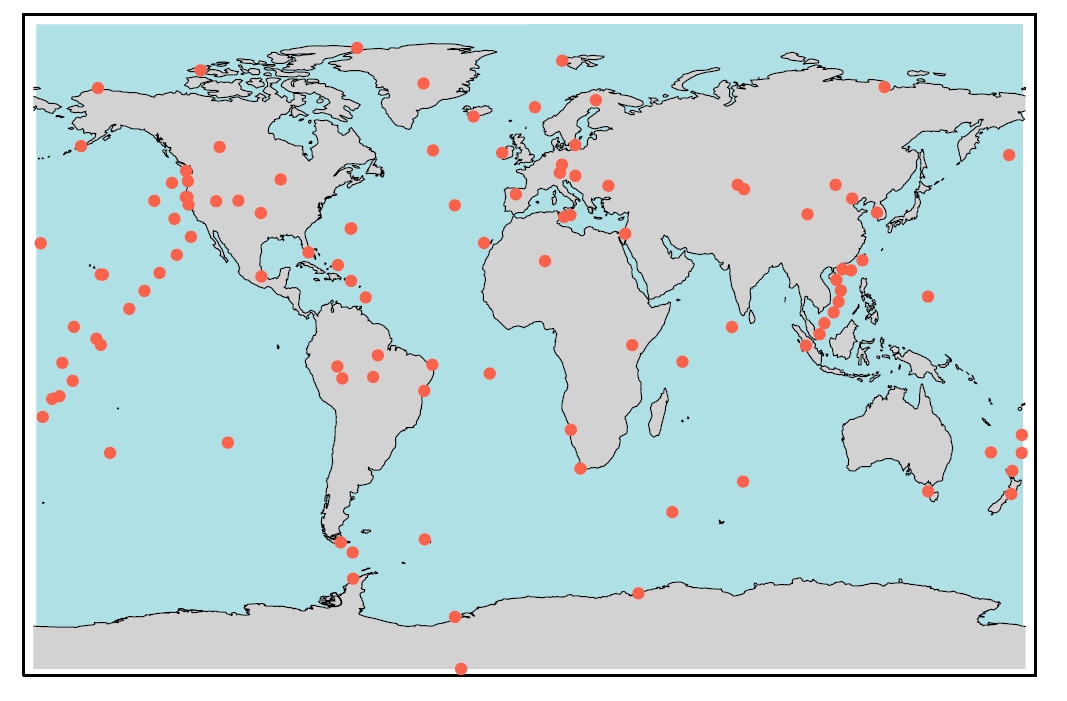


Fig. S1 Locations where CO_2_ mixing ratio data are being measured which are used to estimate CO_2_ surface fluxes using atmospheric transport inversions.


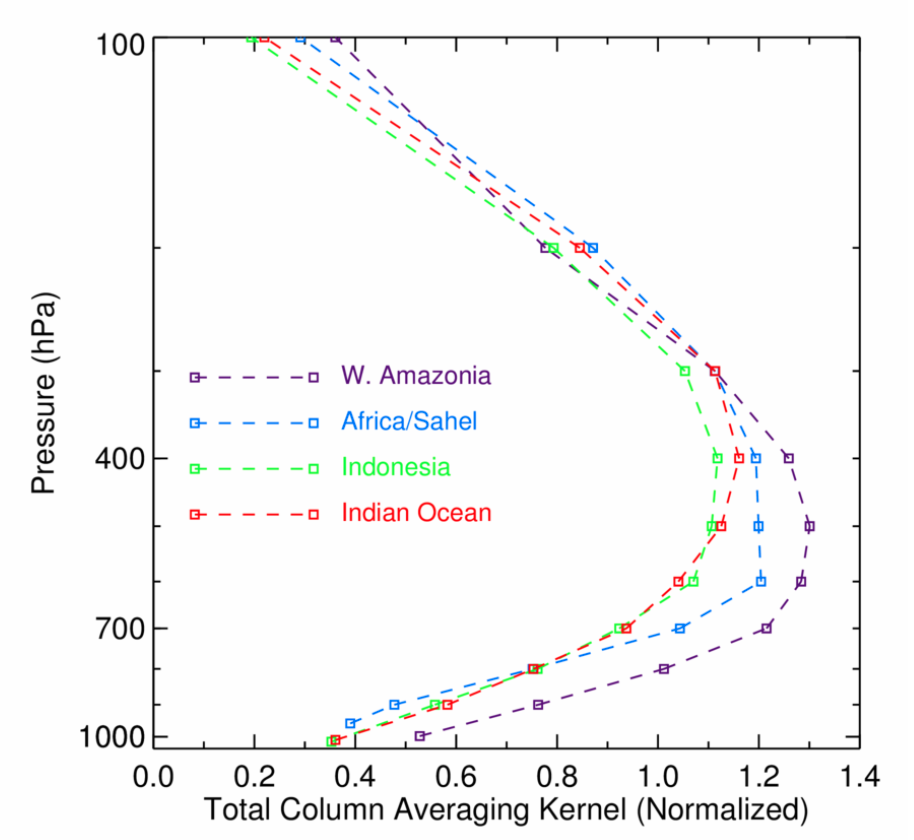


Fig. S5 Example (October 1, 2015) of CO retrieval weighting kernels for total column CO estimation using MOPITT data for 3-by-3 degree latitude by longitude regions in Amazonia, Africa, Indonesia, and the Indian Ocean respectively. The weighting kernels are normalized such that they represent the response of the retrieved total column to a perturbation in CO partial column applied at each level in the retrieved profile.  The ideal value would be 1 at all levels.

**Provided separately**

Fig. S2 Availability of atmospheric CO_2_ data used for atmospheric transport inversions.

Fig. S3 Gravity anomaly anomalies as measured by GRACE satellites for 2015 and 2016.

Fig. S4 Precipitation anomalies calculated from TRMM (Tropical Rainfall Measuring Mission) (Huffman et al. 2007) version 7 and monthly daily maximum and minimum temperature from NOAA Climate Prediction Center (CPC) climatology (Fan et al. 2008).
