# Supplementary figures and images for: Tropical land carbon cycle responses to 2015/16 El Niño as recorded by atmospheric greenhouse gas and remote sensing data
Source: Philos Trans R Soc Lond B Biol Sci. 2018 Oct 8;373(1760):20170302. doi: 10.1098/rstb.2017.0302 (PMC6178440; doi:10.1098/rstb.2017.0302)

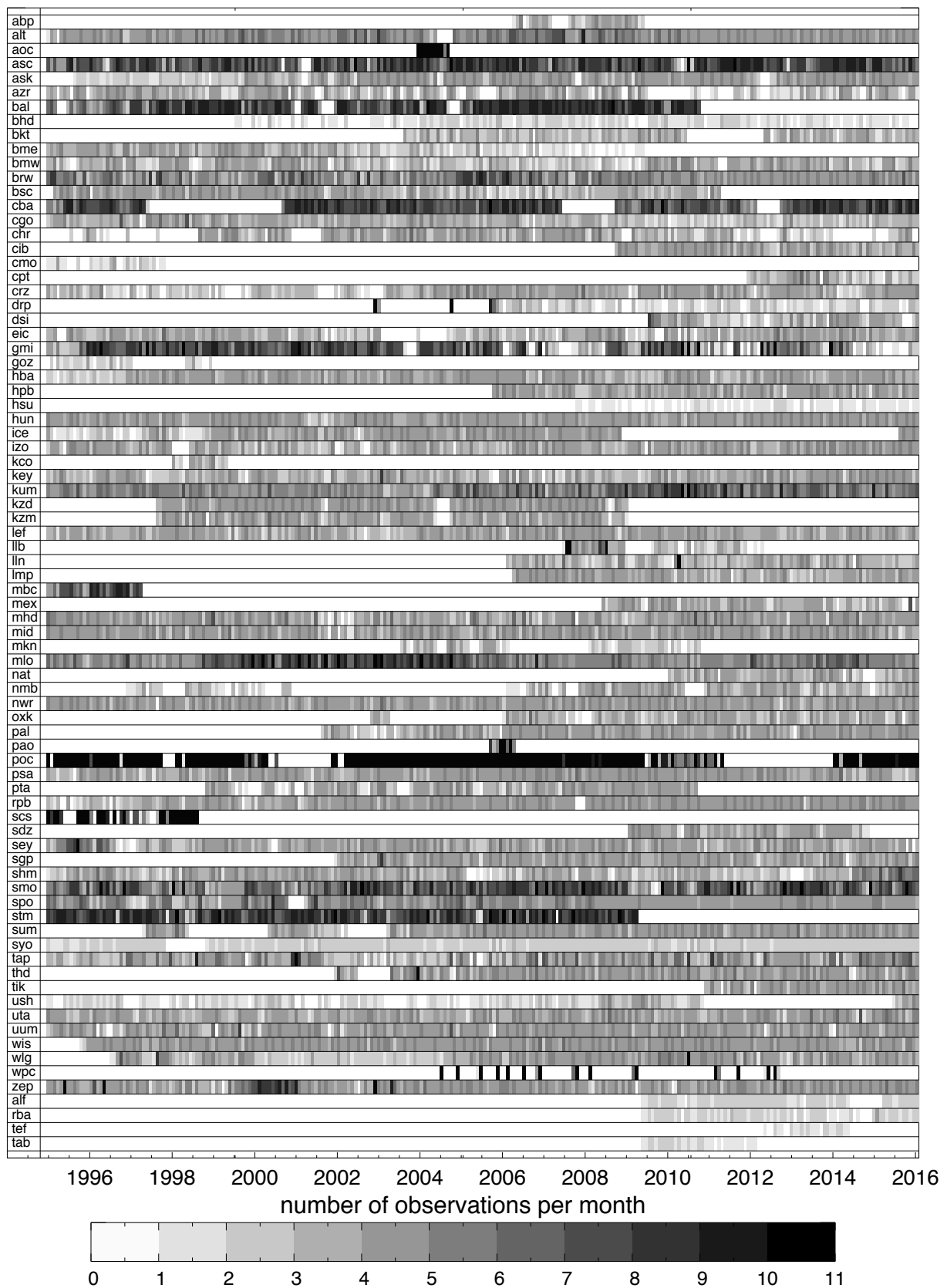

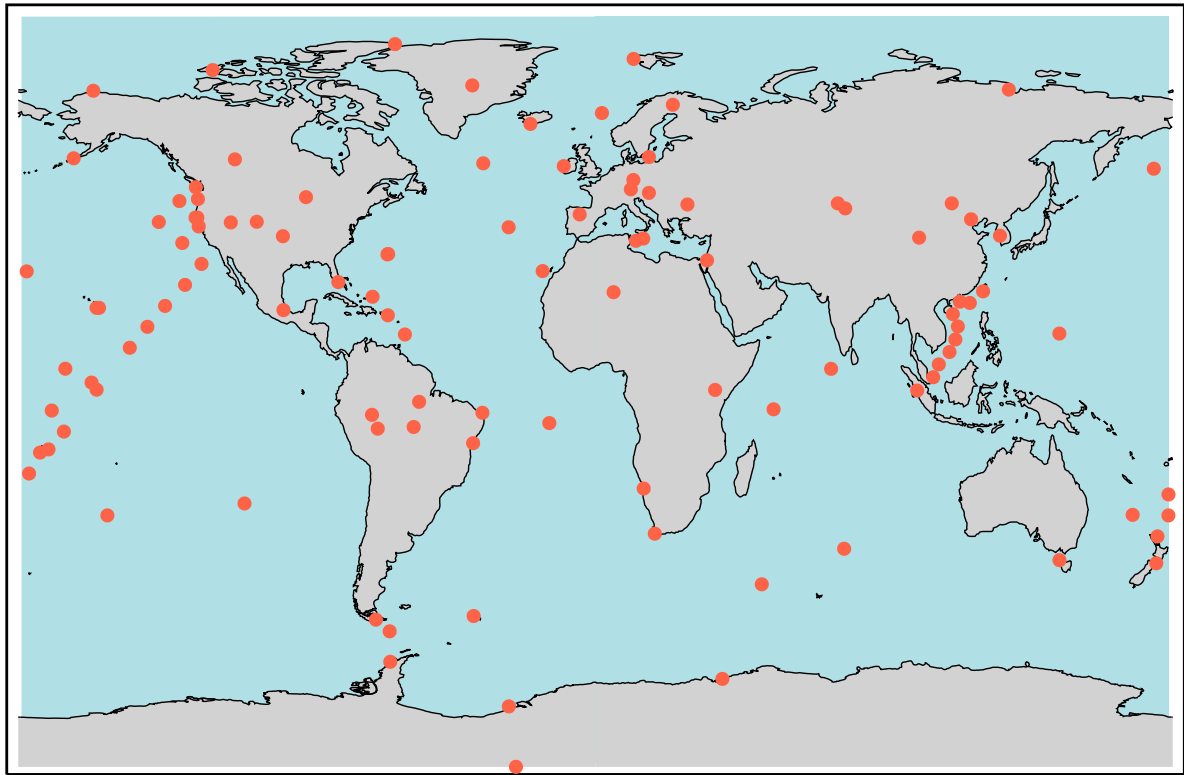

Supplement: Figure S2 [file rstb20170302supp2.pdf]

2015

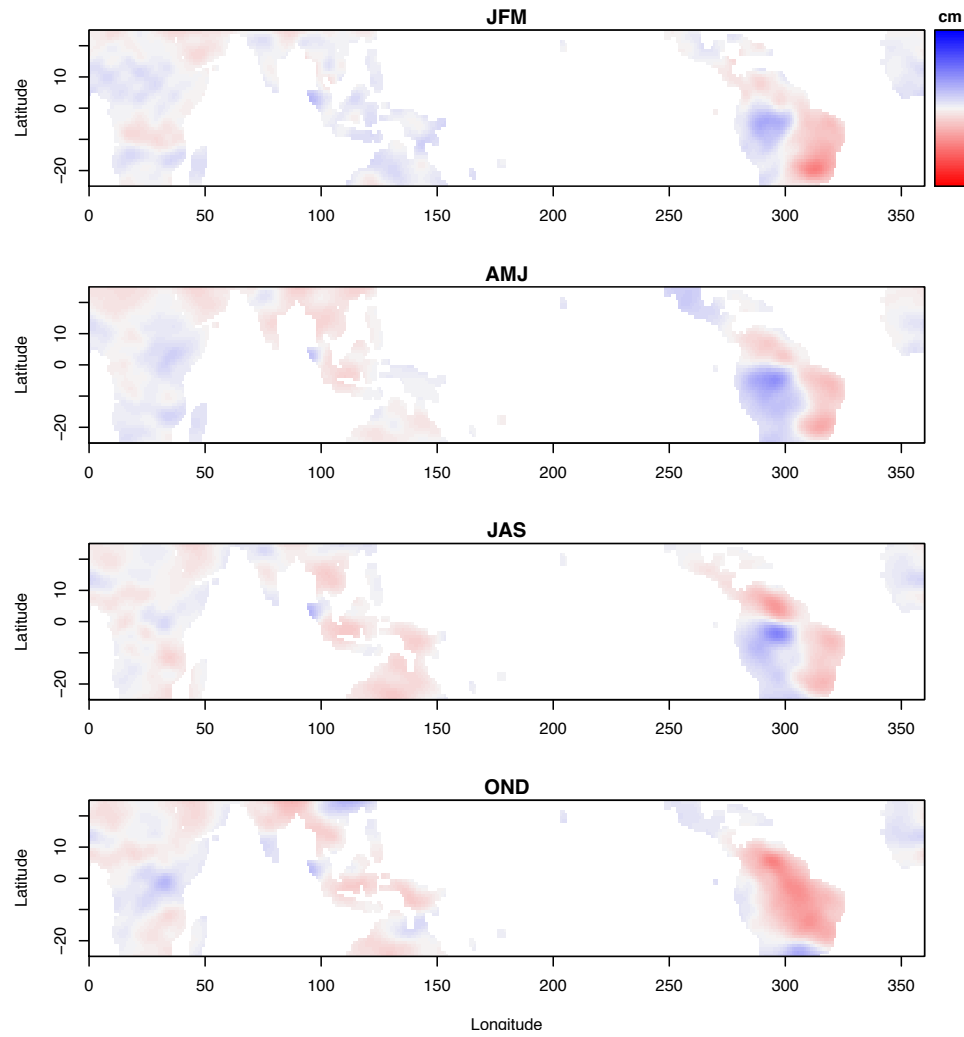

2016

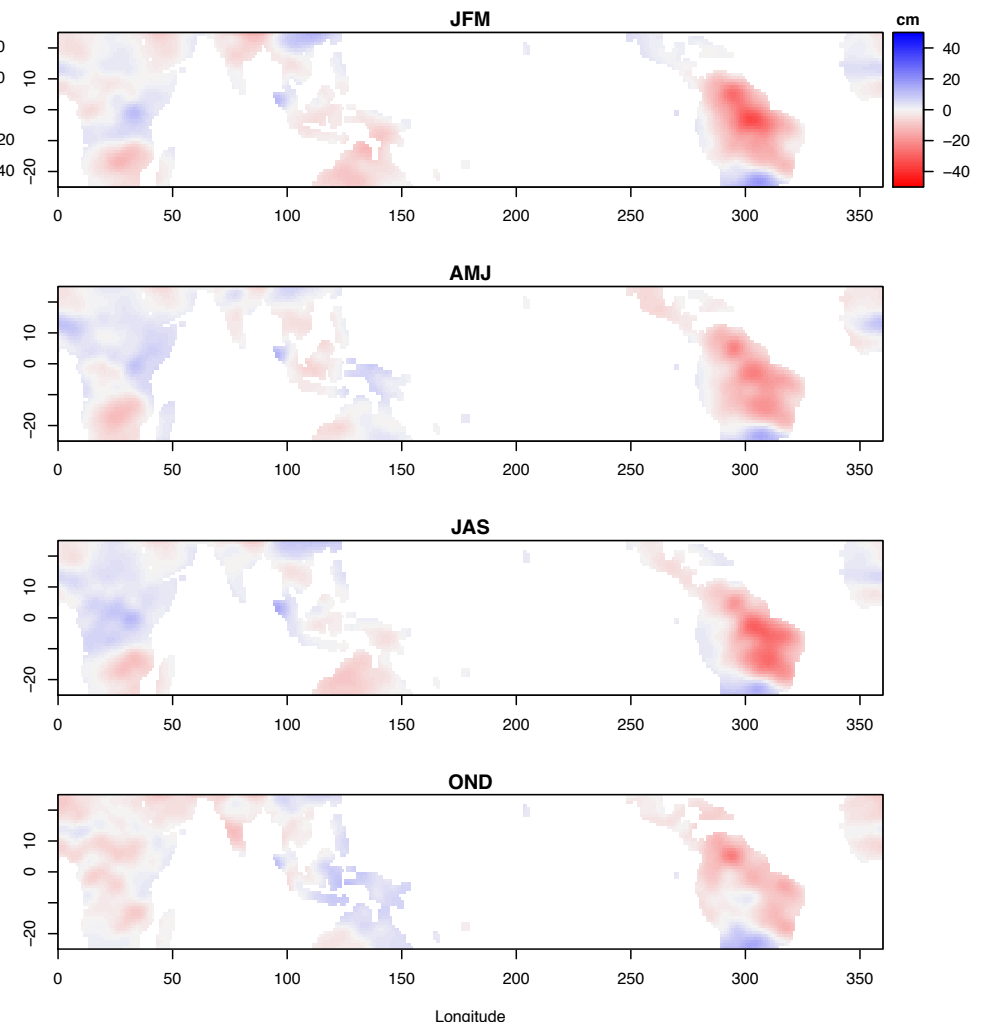

Supplement: Figure S3 [file rstb20170302supp3.pdf]
