# Supplementary material for: Tropical land carbon cycle responses to 2015/16 El Niño as recorded by atmospheric greenhouse gas and remote sensing data
Source: Philos Trans R Soc Lond B Biol Sci. 2018 Oct 8;373(1760):20170302. doi: 10.1098/rstb.2017.0302 (PMC6178440; doi:10.1098/rstb.2017.0302)
Supplement: Figure S4A and B [file rstb20170302supp4.pdf]

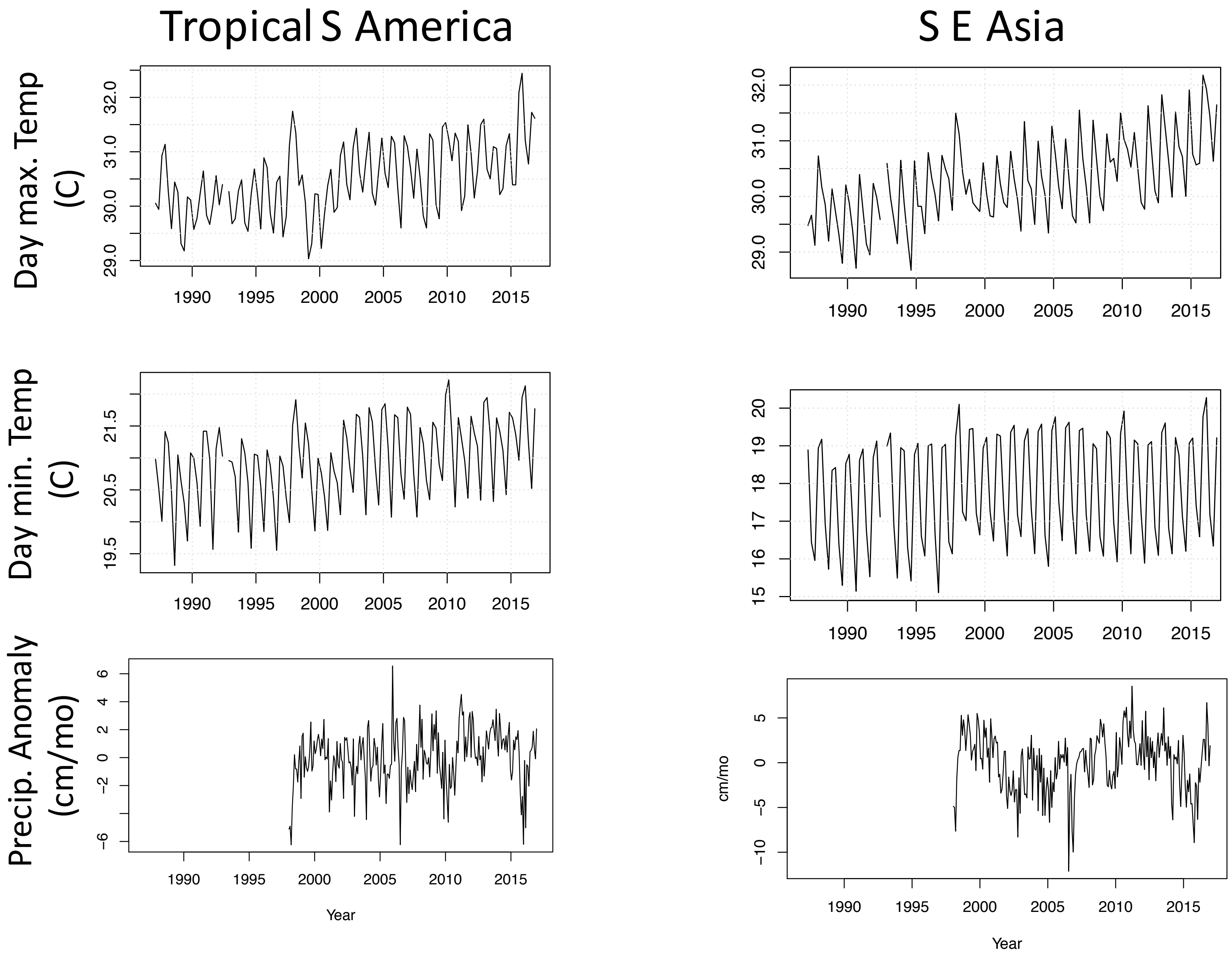

Tropical S America
S E Asia
Day max. Temp (C)
Day min. Temp (C)
Precip. Anomaly (cm/mo)
cm/mo
Year
29.0
30.0
31.0
32.0
19.5
20.5
21.5
15
16
17
18
19
20
−6
−2
0
2
4
6
−10
−5
0
5
1990
1995
2000
2005
2010
2015

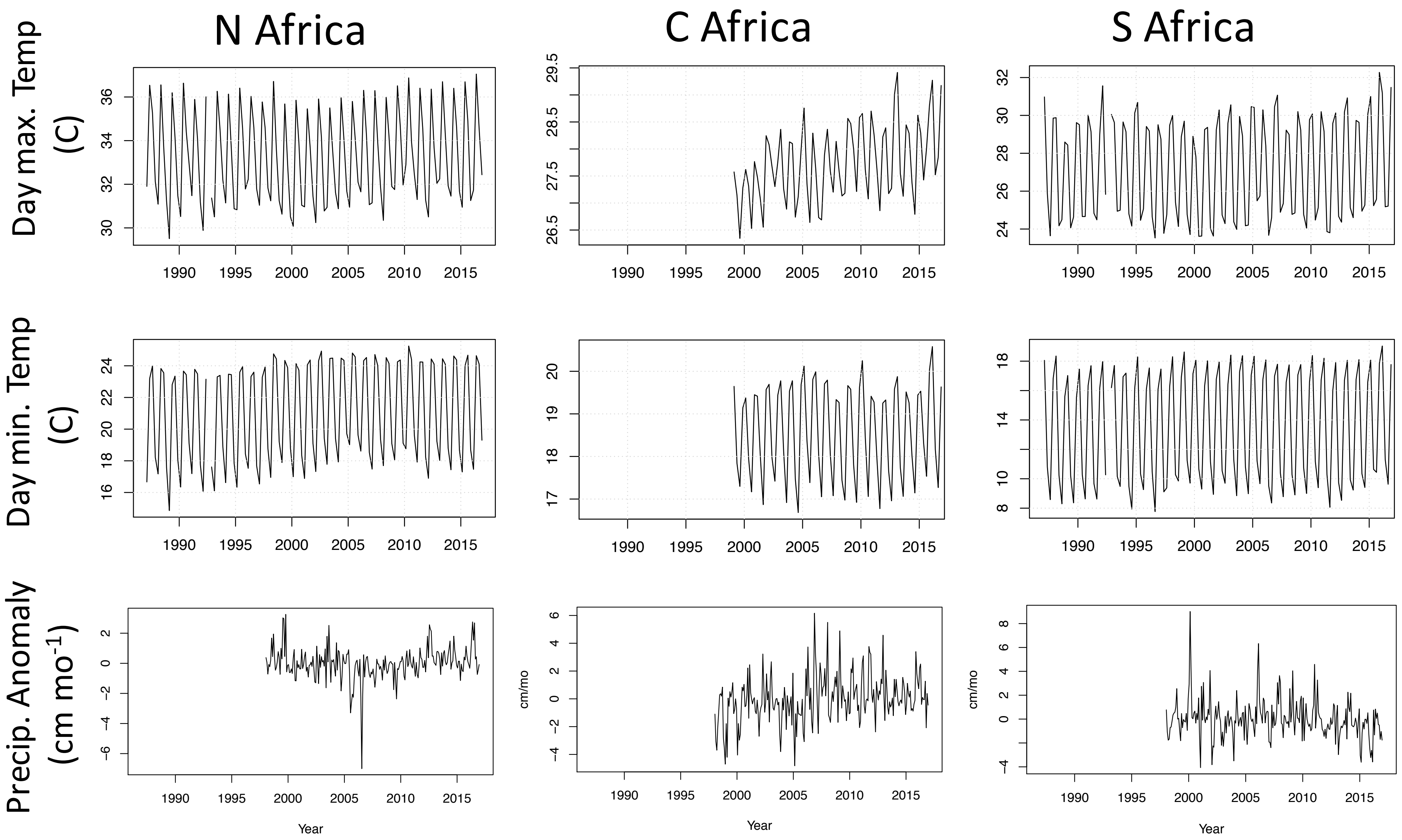

N Africa
C Africa
S Africa
Day max. Temp (C)
Day min. Temp (C)
Precip. Anomaly (cm mo-1)
cm/mo
cm/mo
Year
Year
Year
1990
1995
2000
2005
2010
2015
